# A state of reversible compensated ventricular dysfunction precedes pathological remodelling in response to cardiomyocyte-specific activity of angiotensin II type-1 receptor in mice

**DOI:** 10.1242/dmm.019174

**Published:** 2015-08-01

**Authors:** Georgia A. Frentzou, Mark J. Drinkhill, Neil A. Turner, Stephen G. Ball, Justin F. X. Ainscough

**Affiliations:** Leeds Institute of Cardiovascular & Metabolic Medicine, and Multidisciplinary Cardiovascular Research Centre, University of Leeds, Leeds LS2 9JT, UK

**Keywords:** Renin angiotensin system, Conditional transgenic mouse, Cardiac hypertrophy, Fibrosis, Dysfunction, Remodelling

## Abstract

Cardiac dysfunction is commonly associated with high-blood-pressure-induced cardiomyocyte hypertrophy, in response to aberrant renin-angiotensin system (RAS) activity. Ensuing pathological remodelling promotes cardiomyocyte death and cardiac fibroblast activation, leading to cardiac fibrosis. The initiating cellular mechanisms that underlie this progressive disease are poorly understood. We previously reported a conditional mouse model in which a human angiotensin II type-I receptor transgene (HART) was expressed in differentiated cardiomyocytes after they had fully matured, but not during development. Twelve-month-old HART mice exhibited ventricular dysfunction and cardiomyocyte hypertrophy with interstitial fibrosis following full receptor stimulation, without affecting blood pressure. Here, we show that chronic HART activity in young adult mice causes ventricular dysfunction without hypertrophy, fibrosis or cardiomyocyte death. Dysfunction correlated with reduced expression of pro-hypertrophy markers and increased expression of pro-angiogenic markers in the cardiomyocytes experiencing increased receptor load. This stimulates responsive changes in closely associated non-myocyte cells, including the downregulation of pro-angiogenic genes, a dampened inflammatory response and upregulation of *Tgfβ*. Importantly, this state of compensated dysfunction was reversible. Furthermore, increased stimulation of the receptors on the cardiomyocytes caused a switch in the secondary response from the non-myocyte cells. Progressive cardiac remodelling was stimulated through hypertrophy and death of individual cardiomyocytes, with infiltration, proliferation and activation of fibroblast and inflammatory cells, leading to increased angiogenic and inflammatory signalling. Together, these data demonstrate that a state of pre-hypertrophic compensated dysfunction can exist in affected individuals before common markers of heart disease are detectable. The data also suggest that there is an initial response from the housekeeping cells of the heart to signals emanating from distressed neighbouring cardiomyocytes to suppress those changes most commonly associated with progressive heart disease. We suggest that the reversible nature of this state of compensated dysfunction presents an ideal window of opportunity for personalised therapeutic intervention.

## Abstract (precis)

**Highlighted Article:** A novel conditional mouse model was used to investigate early initiating stages of heart disease that are commonly overlooked, and identifies a ‘window of opportunity’ for personalised therapeutic intervention.

## INTRODUCTION

Heart failure is a progressive condition defined by cellular and molecular abnormalities that lead to pathological remodelling, a key feature of which is cardiomyocyte (CM) hypertrophy (Katz, 1994, 2003). Cardiac hypertrophy is characterised by CM loss, proliferation of cardiac fibroblasts, and collagen deposition (Braunwald and Bristow, 2000; Frey and Olson, 2003), leading to interstitial fibrosis with stiffening of the ventricles and impaired contractile function (Shirwany and Weber, 2006; Weber, 2005). Although various triggers have been identified (Braunwald and Bristow, 2000; Frey and Olson, 2003), the mechanism behind switching from normal state to hypertrophy and remodelling remains enigmatic. Of particular interest is the renin-angiotensin system (RAS). Angiotensin II (AngII), the major active component of RAS, is responsible for regulating arterial blood pressure, plasma volume and sympathetic nervous activity, and can also promote growth and proliferation through activation of signalling mechanisms (Berry et al., 2001; Frey and Olson, 2003; Levy, 2005; Oro et al., 2007) primarily through the type-1 receptor (AT1R).

RAS activity is involved in heart failure beyond its influence on blood pressure, because selective blockade attenuates hypertrophy (Barrios et al., 2007; Okin et al., 2003). Furthermore, AT1R can be stimulated locally (Zou et al., 2004) and could thus promote localised growth. Owing to the ubiquitous nature of RAS, separating systemic from localized activity has been difficult. Previously, we reported a novel model of heart disease that is highly controllable, the human angiotensin receptor transgenic (HART) mouse, in which *in utero* effects of receptor stimulation are avoided and expression is induced in adulthood (Ainscough et al., 2009). In the unstimulated state the transgenic receptors exhibit low-level activity confined to CM cells, whereas stimulation with the AngII byproduct, AngIV, confers full activity. Twelve-month-old HART mice developed cardiac hypertrophy without fibrosis under unstimulated conditions, whereas stimulation with AngIV for 4 weeks exacerbated the hypertrophic response and induced fibrosis. Hearts from these aged HART mice also exhibited dilatation with reduced ejection fraction. Importantly, these changes occurred in the absence of changes in blood pressure. We therefore hypothesized that this controllable model of cardiac remodelling is uniquely suited to dissecting early progressive changes before the onset of hypertrophy and initiation of fibrosis.

Here, we have identified and characterised a previously unreported state of heart failure prior to the development of hypertrophy, which we term pre-hypertrophic compensated heart failure. We utilised our HART model to investigate initiating events in young adult mice, with and without stimulation of CM-specific receptors by AngIV. Our results demonstrate that AT1R activity drives CM dysfunction prior to hypertrophy or fixed myocardial remodelling. Furthermore, we show that this correlates with enhanced CM-specific pro-angiogenic gene expression, and a concomitant and more significant decrease from the non-myocyte (NM) population. Changes occurring in the CM population were associated with a responsive wave of changes in the NM population of the heart, primarily downregulation of inflammatory markers and upregulation of *Tgfβ*. Subsequent stimulation of the receptors with AngIV resulted in further cardiac dysfunction, CM hypertrophy, and initiation of fibrosis. This step change in the remodelling process correlated with further (albeit subtle) changes in both CM and NM populations, notably increased inflammatory markers from NM cells. At no time during the switch from ‘normal’ to ‘compensated’ and then to ‘progressive’ remodelling did we detect significant alterations in collagen, expressed exclusively by the NM population. This investigation therefore unveils a complex interplay between different cellular compartments of the heart during very early initiating events of pathological remodelling in response to CM-specific AT1R-induced cellular dysfunction.

**TRANSLATIONAL IMPACT**

**Clinical issue**

Poor heart function (heart failure) is usually associated with enlargement of heart muscle cells induced by high blood pressure, in response to altered renin-angiotensin system (RAS) activity throughout the body. Subsequent complications include the death of heart muscle cells and increased production and activation of fibroblasts resulting in fibrosis, stiffening, and worsening ability for the heart to pump effectively. Increasing evidence suggests, however, that RAS activity within individual heart muscle cells plays a direct role in this process, independent of blood pressure, but this has been very difficult to resolve. Consequently, the blood-pressure-independent mechanisms that regulate the early stages of heart failure remain unknown. A better understanding of these mechanisms would facilitate development of personalised treatments against this debilitating disease.

****Results****

Here, the authors use a transgenic mouse model to investigate the initiating stages of heart failure. In the model, expression of a human angiotensin II type-I receptor transgene (HART) is turned on in heart muscle cells after they have fully formed. At 5 months old, the hearts of HART mice have reduced pumping ability without muscle cell enlargement or death, or fibroblast activation. Importantly, this stage is completely reversible simply by inhibiting the production of the angiotensin receptors. Reduced heart function in the HART mice correlates with reduced expression in heart muscle cells of genes involved in the regulation of blood pressure, salt level, calcium handling and mitochondrial function. Moreover, other cells of the heart show suppression of genes involved in the production of new blood vessels and in inflammatory responses. Further activation of the receptors, however, causes enlargement and sporadic death of the heart muscle cells, with increased production and activation of fibroblasts and inflammation-associated cells, signalling onset of progressive deterioration.

****Implications and future directions****

This study identifies a state of compensated heart dysfunction that occurs in response to increased RAS activity within individual heart muscle cells. The condition seems to be remarkably stable and, more importantly, treatable with RAS inhibitors, before any long-term damage is initiated. An appropriate screening procedure could be adopted, therefore, to identify ‘at risk’ patients with reduced heart function but no other signs of progressive heart disease. A personalised treatment regimen for these patients with available RAS inhibitors, and regular monitoring of heart function, might prevent the progressive stages of heart failure occurring by intervening before the disease has had the chance to properly take hold.

## RESULTS

The primary reason for this study was to investigate the initiating mechanisms and complex interplay between different cell types of the heart that occur during the earliest stages of progressive heart disease, with a view to revealing accessible targets for development of novel therapeutics. We have used the HART activation system in 5-month-old males to relate phenotypic changes that occur during the switch from normal to remodelling state with changes in gene expression specific to the CM and NM populations of the heart.

### HART activity induces a reversible state of pre-hypertrophic compensated dysfunction

**Fig. 1.**
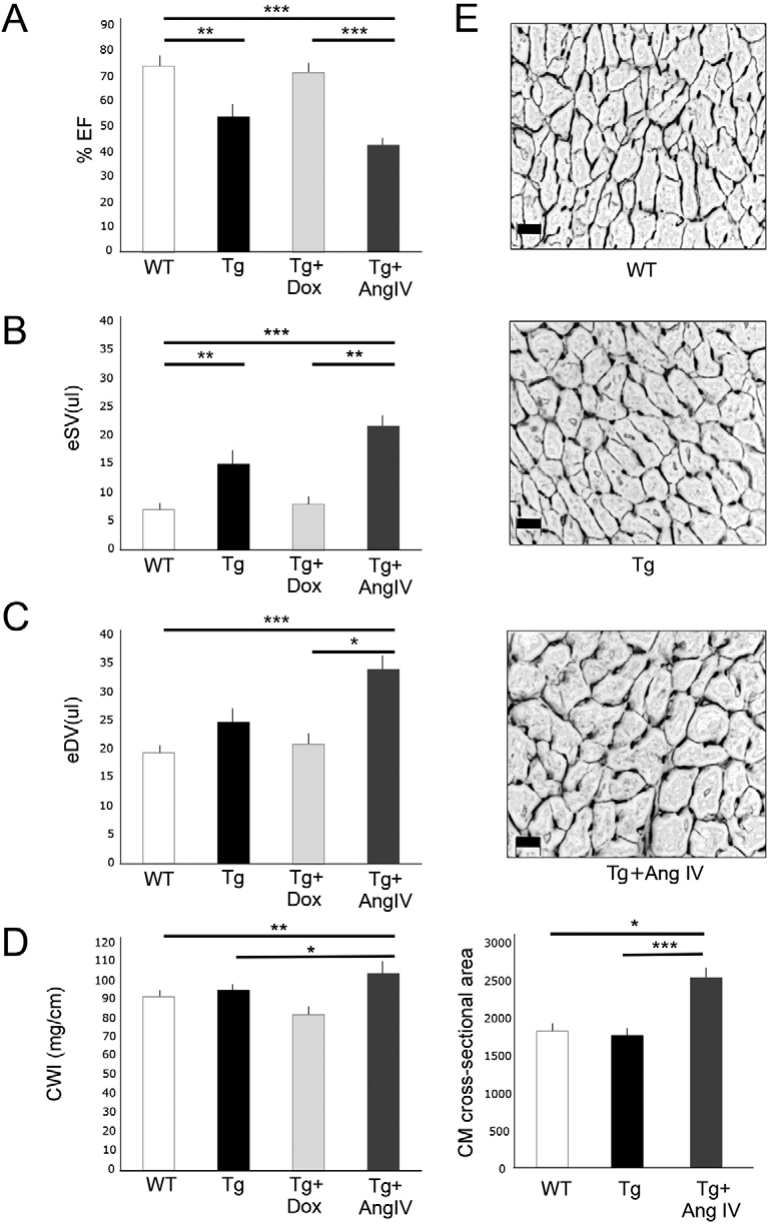
**Ventricular dysfunction is observed in 5-month-old HART mice prior to onset of hypertrophy or fibrosis.** Stimulation with AngIV for 4 weeks promotes hypertrophy with worsening ventricular dysfunction, whereas inhibition of receptor expression improves function. (A) Ejection fraction (%EF) of HART males (Tg) was significantly reduced relative to wild-type littermate controls (WT). Exposure to AngIV caused further reduction in EF, whereas Dox treatment relieved the dysfunction. (B,C) End systolic volume (eSV) and end diastolic volume (eDV) were significantly increased in Tg males, and further increased following exposure to AngIV, consistent with cardiac dilatation. Dox treatment reduced eSV and eDV to WT levels within 8 weeks. (D) Cardiac weight index (CWI) for WT and Tg mice showed no significant change, whereas AngIV treatment induced a significant increase. (E) Representative fields of view of sectioned left ventricle showing CM population in cross-section. Cell membranes and ECM were detected with WGA, and nuclei with Hoechst 33342. CM cross-sectional area was similar between WT and Tg mice, and significantly increased following AngIV stimulation (*n*≥4 mice per group, 5 fields of view per mouse). Scale bars: 20 µm. All data are mean±s.e.m. Degree of significance between groups is indicated (*P≤0.05, **P≤0.01, ***P≤0.001).

Five-month-old wild-type (WT) and transgenic (Tg) HART mice, in which AT1R was overexpressed exclusively in differentiated CM cells, were analysed by Millar catheter. Ejection fraction (EF) was significantly decreased in Tg compared to WT mice (Table 1, Fig. 1A), whereas end systolic volume (eSV) and end diastolic volume (eDV) were increased (Table 1, Fig. 1B,C). When HART mice were reintroduced Dox at 4 months, to block production of new transgenic receptors, Millar catheter analysis showed significant improvement in ventricular function, approaching WT levels (Table 1, Fig. 1A-C). After 8 weeks of Dox treatment (Table 1, Group G), EF, eSV and eDV were indistinguishable from normal (Group A). In contrast, 4 weeks of AngIV treatment caused a further decrease in EF compared to WT and Tg groups, correlating with a significant increase in eSV and eDV, indicative of ventricular dysfunction and dilatation (Table 1, Fig. 1A-C). Previously we showed that the dose of AngIV that is infused does not influence blood pressure (Ainscough et al., 2009). As expected, this dose also had no impact on EF in WT mice (Table 1, Group H). It did, however, cause a notable increase in eDV, suggesting a dynamic role to regulate blood supply that is within normal physiological parameters. We also investigated morphological indicators of ventricular hypertrophy. Cardiac weight index (CWI) remained unchanged between WT and Tg animals, but significantly increased after AngIV treatment (Fig. 1D). Consistent with this, CM cross-sectional area was increased only after AngIV treatment (Fig. 1E).

**Table 1.**
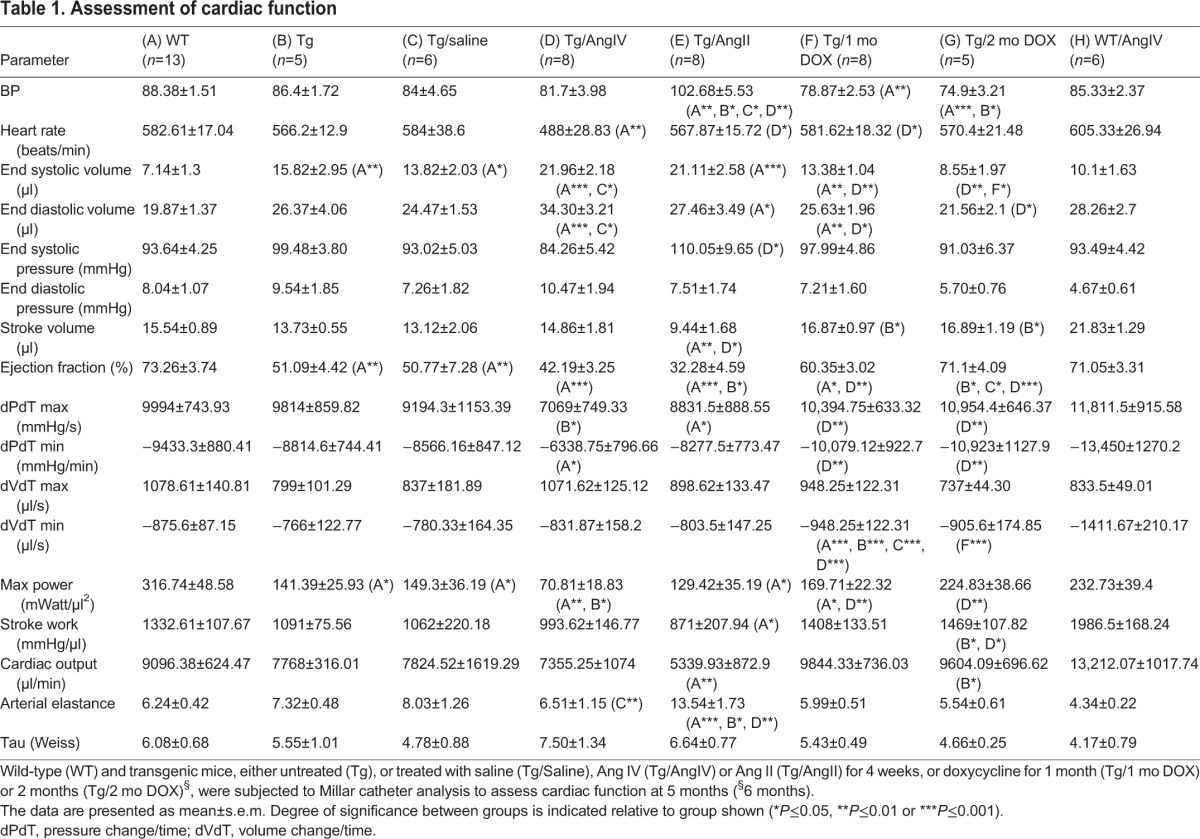
**Assessment of cardiac function**

**Fig. 2.**
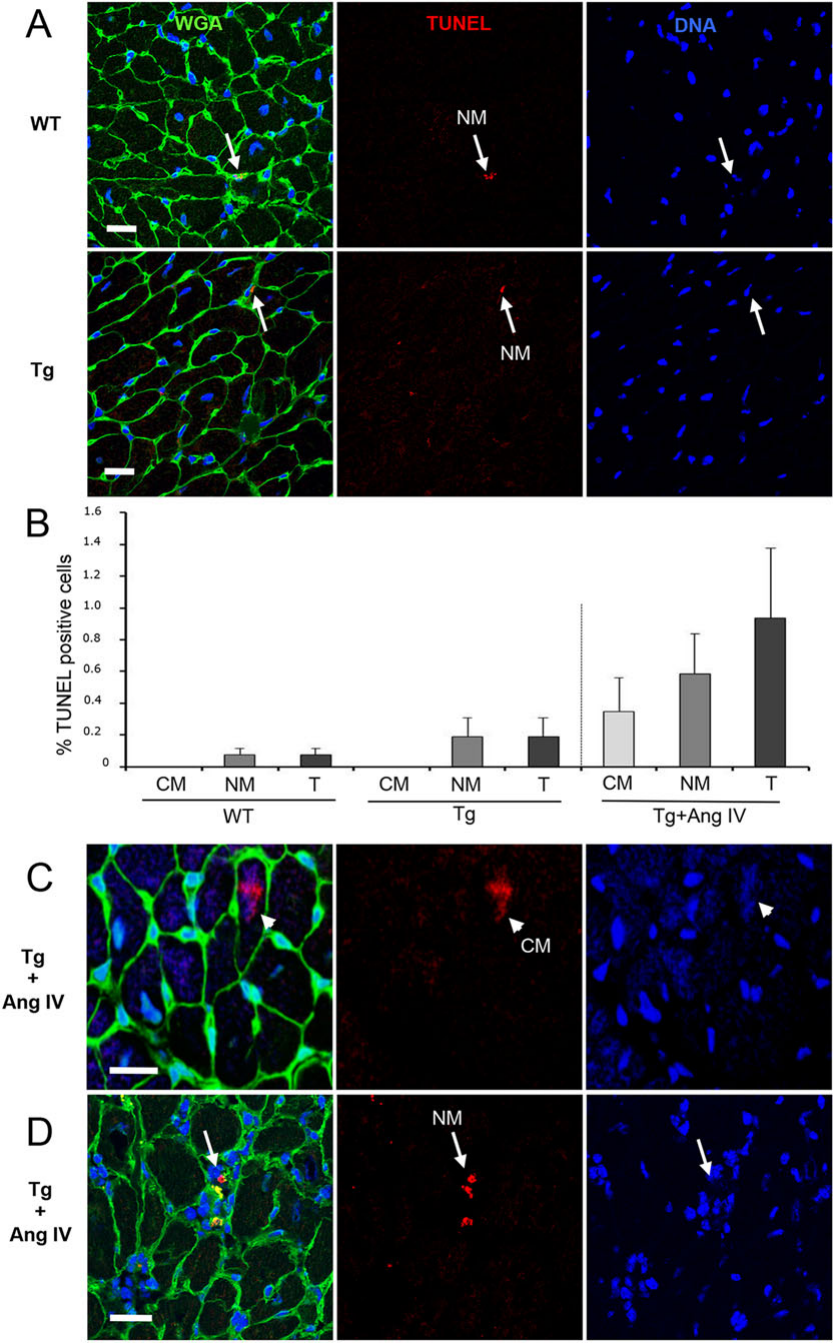
**Stimulation with AngIV for 4 weeks induces cell death in both CM and NM cells of 5-month-old HART mice, promoting fibrosis.** Representative images shown are stained with WGA (green) to detect cell membranes and fibrotic patches, TUNEL (red) to detect fragmenting DNA, and Hoechst 33342 (blue) to detect nuclei. (A) No difference in TUNEL staining, either qualitative or quantitative, was detected between WT and Tg hearts. Occasional positive nuclei (arrows) were detected tightly embedded between CM cell membranes, suggestive of NM status. No CM nuclei were observed to be TUNEL positive. (B) Quantitative analysis of TUNEL data expressed as % positive cells in CM, NM and total cell (T) populations. The number of TUNEL-positive CM cells increased from 0% to 0.4% with AngIV treatment, whereas TUNEL-positive NM cells increased from 0.2% to 0.6%. T increased from 0.2% (Tg) to 0.8% (AngIV), representing a 400% increase in total rate of cell death. (C) Representative example of an AngIV-induced TUNEL-positive CM nucleus (arrowhead) within a healthy region of the myocardium. (D) After AngIV treatment, TUNEL-positive NM nuclei were frequently observed in clusters embedded within areas of developing fibrosis (arrow). Scale bars: 20 µm.

We next looked for evidence of fibrosis. Previously we showed that, in addition to labelling of cell membranes, wheat germ agglutinin (WGA) can efficiently detect very small regions of developing fibrosis (Ainscough et al., 2009). Conventional staining techniques were less reliable unless the fibrotic lesions were advanced. No major fibrotic lesions were observed in either WT or Tg hearts (Fig. 2A). We also detected minimal evidence, in either WT or Tg hearts, for cell turnover modulated by apoptosis and/or necrosis, using TUNEL as a marker of DNA fragmentation (Fig. 2A). Rare TUNEL-positive cells observed in both WT and Tg hearts were confined to the NM population (Fig. 2B). Immunostaining for cleaved caspase-3 as a secondary marker of cell death confirmed these findings, with no evidence for positive staining in either WT or Tg myocardium (supplementary material Fig. S1). In contrast to WT and Tg animals, WGA staining of Tg hearts after AngIV infusion revealed sporadic fibrotic lesions within the left ventricle, with induction of CM cell death (Fig. 2C) in addition to NM cell death (Fig. 2D) in otherwise healthy regions of the Tg myocardium. TUNEL signal was evident in approximately 0.4% of the CM population, whereas cell death in the NM population increased over threefold (Fig. 2B). However, no evidence was observed for induction of cleaved caspase-3 in these hearts (supplementary material Fig. S1).

**Fig. 3.**
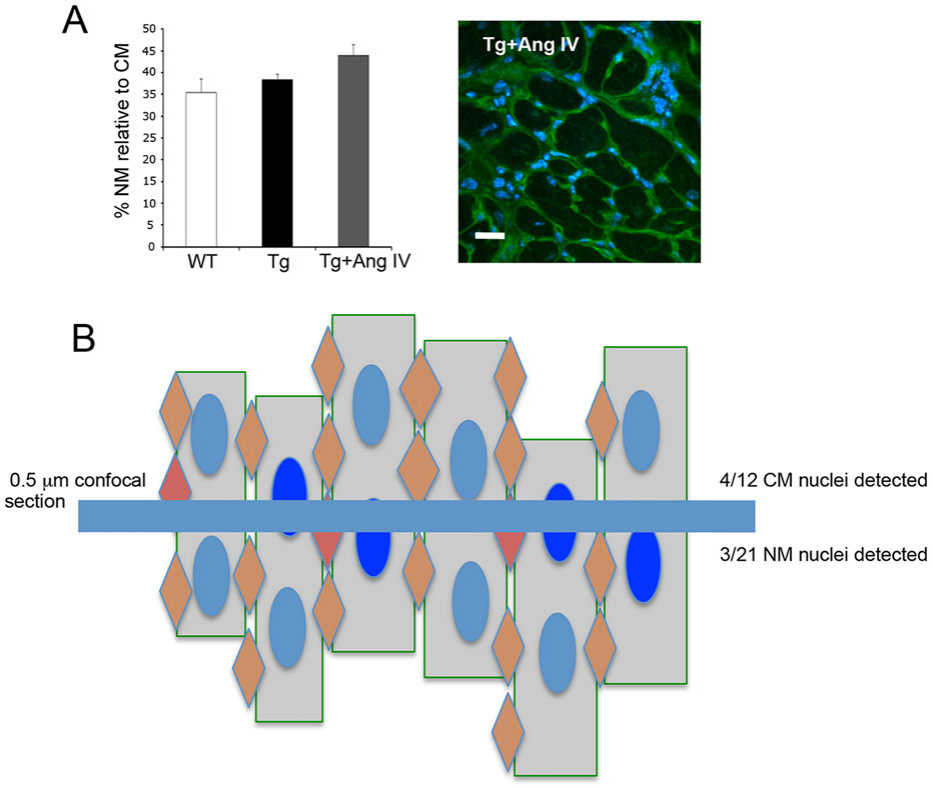
**The ratio of CM:NM cells in healthy regions of the heart changes in response to AngIV treatment.** (A) The proportion of NM cells in healthy regions was increased in Tg hearts following AngIV treatment. The image shows, additionally, that small clusters of NM cells congregate within areas of developing fibrosis in ‘unhealthy’ regions of the myocardium. Scale bar: 20 µm. (B) Schematic representation depicting (blue line) the area of ‘visibility’ within each section image in relation to the three-dimensional structure of the myocardium. The number of nuclei detected is a vast under-representation of the true number of cells; thus, the ratio presented in panel A effectively under-represents the number of NM cells by approximately five- to ten-fold.

Consistent with the CWI, fibrosis, TUNEL and cleaved caspase-3 data, no change was detected in the relative proportion of NM to CM cells, with both WT and Tg hearts exhibiting between 35-40% NM cells per 0.5 µm section depth (Fig. 3A). Moreover, the AngIV-treated group showed evidence for increased NM proliferation (most likely fibroblasts and/or inflammatory cells), seen as clusters of NM nuclei interspersed between groups of CM cells (Fig. 3A). It is worth noting that CM cells are approximately 5-10× longer than NM cells (∼100 µm cf. 10-20 µm); thus, the ratio of NM:CM cells reported here likely under represents the true situation (Fig. 3B).

**Fig. 4.**
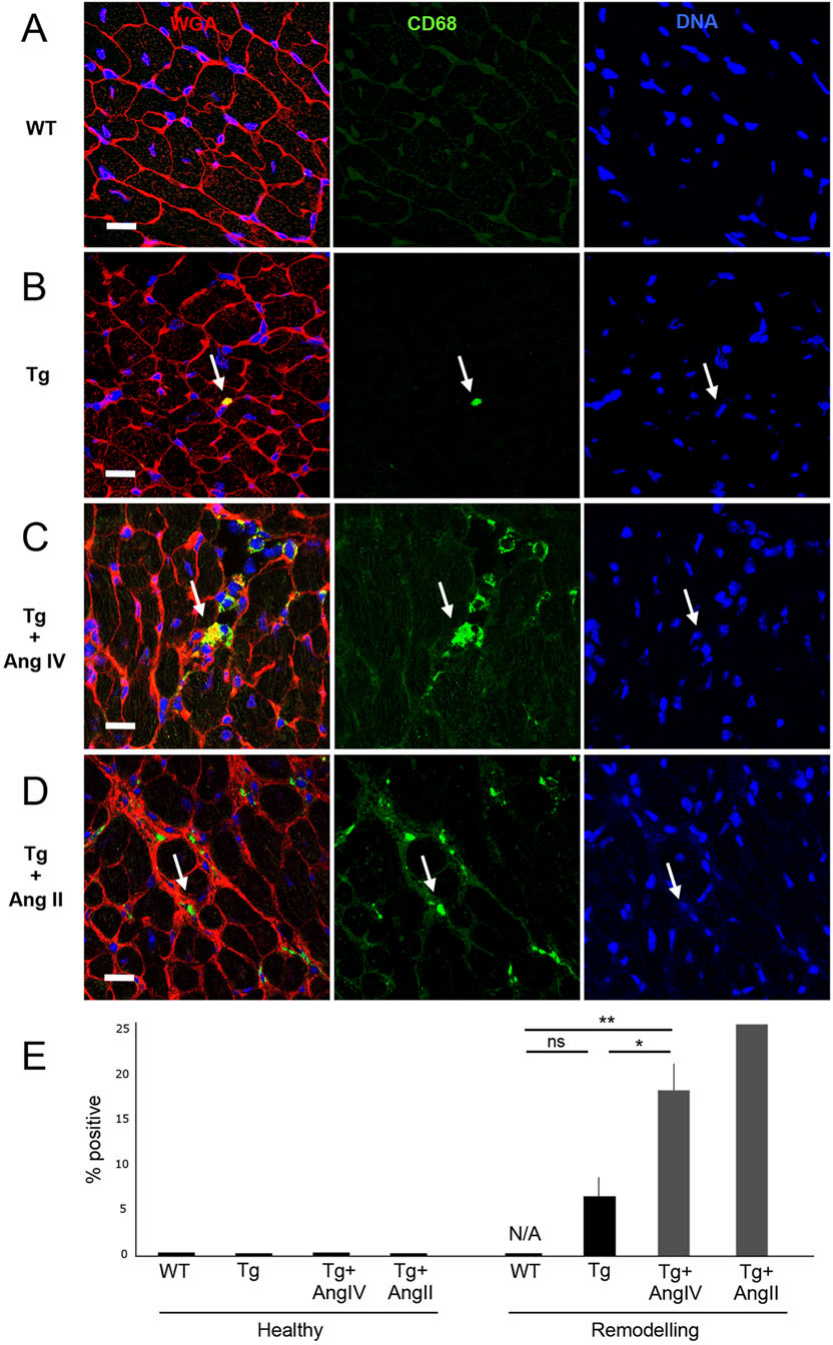
**Stimulation with AngIV for 4 weeks promotes macrophage infiltration.** Representative images shown are stained with WGA (red) to detect cell membranes and fibrotic patches, CD68 (green) to detect macrophages, and DAPI (blue) to detect cell nuclei. (A) No CD68-positive cells were detected in WT hearts. (B) Sporadic single positive cells were detected in Tg hearts, but were not significantly different to WT. (C) Treatment with AngIV resulted in an increased number of CD68-positive cell clusters embedded within areas of developing fibrosis (arrow). This was comparable to HART hearts treated with AngII (D), which exhibited more fibrosis. As expected, the only positive cells were detected tightly embedded between CM cell membranes. Scale bars: 20 µm. (E) Quantitation of CD68 data presented as % positive cells in ‘healthy’ and ‘remodelling’ areas of myocardium. Macrophages were only observed in regions undergoing initiation of remodelling. The proportion of ‘remodelling’ myocardium was increased in Tg hearts after AngIV and AngII treatment, but were still low at the level of the whole myocardium. Degree of significance between groups is indicated (*P≤0.05; **P≤0.01; ns, not significant).

We next looked for evidence of increased inflammation that might correlate with the observed dysfunction, using the macrophage-specific marker CD68 (Fig. 4). Whereas no CD68-positive cells were detected in WT hearts, sporadic individual positive cells were observed in Tg hearts at a low frequency (Fig. 4A,B,E). In contrast, AngIV treatment led to a significant increase in macrophage number, exclusively clustered in regions undergoing fibrotic remodelling (Fig. 4C,E). To assess the extent of AngIV-induced remodelling, we subjected a further cohort to treatment with AngII, which stimulates AT1 receptors in all cells (not just CM) and induces pressure overload (Table 1). This caused more extensive fibrosis (not shown), with Millar catheter analyses indicating more severe heart failure as evidenced by reduced EF (Table 1). AngII-treated myocardium showed evidence of significant macrophage infiltration clustered within zones of remodelling similar to that in the AngIV group (Fig. 4D,E).

Together, these data suggest that low-level chronic AT1 receptor activity on CM cells, without stimulating receptors on other cell types of the heart, is sufficient to cause cardiac dysfunction without fibrosis or induction of a significant inflammatory or apoptotic response. Increased stimulation leads to initiation of sporadic cell death and associated macrophage infiltration. We conclude that the observed cardiac dysfunction is a stable pre-hypertrophic compensated state (PHCS) that is intrinsic to the CM itself, driven directly via low-level signalling from the HART receptors. Importantly, the data shows that cardiac dysfunction during PHCS is reversible and represents an ideal opportunity for therapeutic intervention.

To provide a clearer understanding of how these changes promote the path towards heart failure, we next investigated changes that occur in the CM cells, and secondary responses to these changes in NM cells, separately.

### AngIV modulation of genes associated with late-stage cardiac remodelling

**Fig. 5.**
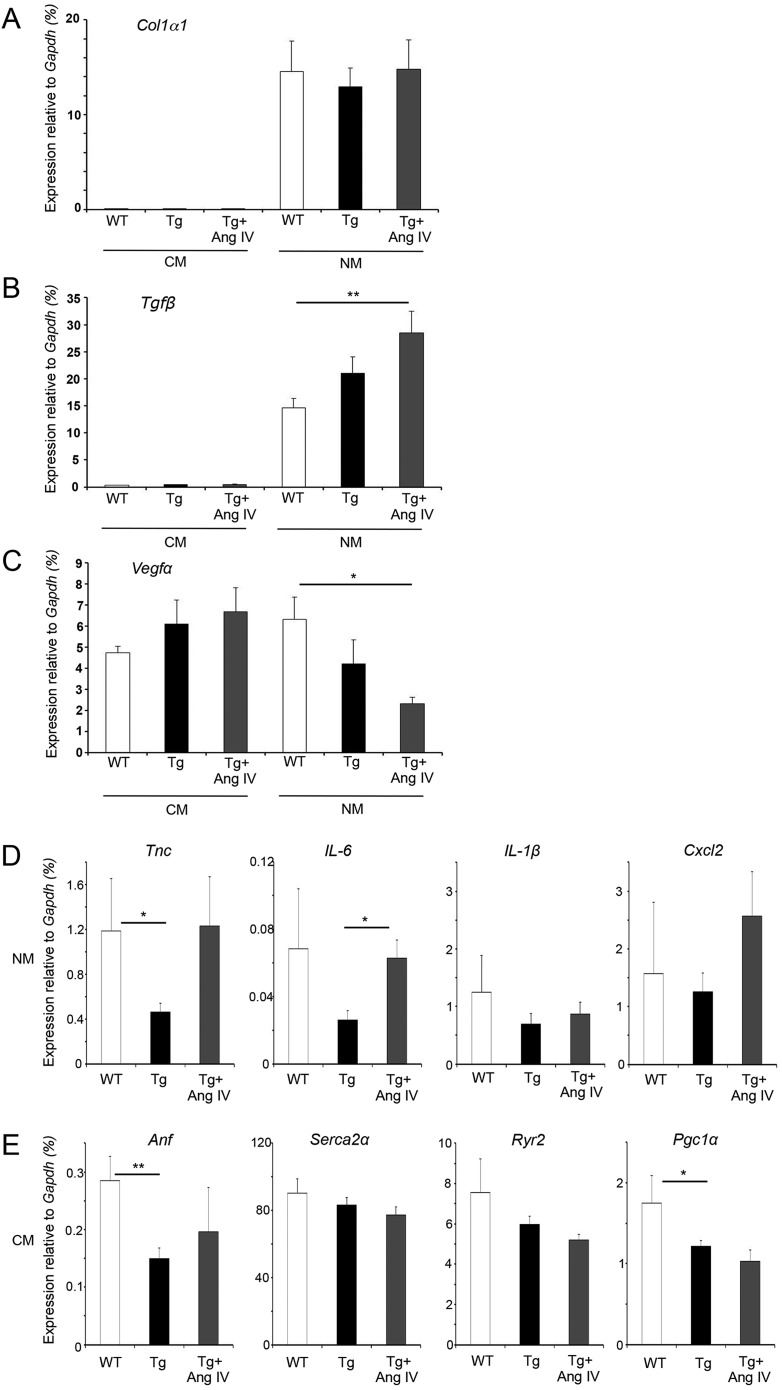
**Remodelling-associated genes are differentially expressed in CM and NM cells, with and without stimulation with AngIV.** (A) *Col1α1* was not expressed in CM cells of any group, whereas NM cells expressed *Col1α1* at similar levels in all groups. (B) *Tgfβ* was also not expressed in CM cells from any group. However, NM cells from Tg mice showed increased *Tgfβ*, with further increase upon AngIV treatment. (C) *Vegfα* was expressed in both CM and NM cells of all groups. Tg CM cells expressed *Vegfα* at higher levels than WT CM cells, with additional increase upon stimulation with AngIV. Conversely, *Vegfα* expression from NM cells was reduced in Tg animals, and further reduced upon AngIV treatment. (D) Pro-inflammatory gene expression signature is dampened in Tg NM cells, but begins to increase upon treatment with AngIV. Expression of the inflammatory marker *Tnc* was significantly decreased in Tg NM cells, but returned to normal levels following AngIV treatment. The pro-inflammatory cytokine *IL-6* was also reduced in Tg NM cells, and significantly increased on exposure to AngIV. *IL-1β* followed a similar profile to *IL6*, albeit less pronounced, whereas the pro-inflammatory chemokine *Cxcl2* was expressed at similar levels in WT and Tg NM cells, and increased upon AngIV treatment. (E) Differential expression of CM-specific genes commonly associated with cardiac dysfunction. *Anf* expression was significantly decreased in the Tg CM population and increased upon treatment with Ang IV. *Serca2α*, *Ryr2* and *Pgc1α* showed a tendency to decrease in Tg CM cells. This response was enhanced upon AngIV treatment. Degree of significance between groups is indicated (*P≤0.05, **P≤0.01).

To investigate the mechanisms and cellular origin behind the observed dysfunction followed by induction of cardiac enlargement and fibrosis, we isolated two populations of cells from 5-month-old hearts: (i) CM and (ii) NM (fibroblasts, vascular and inflammatory cells). Rigorous assessment of cell fraction purity by both microscopy and molecular analyses demonstrated that the CM fraction was almost completely clear of NM contamination, whereas the NM fraction routinely contained a low level of contaminating CM cells (see Materials and Methods, and supplementary material Fig. S2). The transcription factor *GATA4* was expressed at similar levels in both fractions, whereas CM-specific *αMHC* levels were high in the CM fraction and low in the NM fraction. This low level of *αMHC*, most likely caused by inclusion of CM debris from fragmented cells, was routinely used as a marker for purity assessment. *Gapdh* was used as a control because its levels (CT values relative to input cDNA) were consistent between CM and NM populations, and between WT, Tg and Tg+AngIV samples. In contrast, β-actin mRNA levels showed dramatic differences between groups (not shown). Validated high-quality fractions were then used to analyse expression profiles of key genes commonly used as markers of ventricular fibrosis, hypertrophy and angiogenesis (Fig. 5). Supplementary material Fig. S3 shows the same expression data after adjustment to take account of the relative contribution of CM and NM cells, as indicated in Fig. 3A.

Initially, we examined *Col1α1*, which is upregulated in cardiac fibroblasts in response to myocardial damage. As expected, the isolated CM fraction did not express *Col1α1* (Fig. 5A). Importantly, this confirmed that the cell fractionation method generated a pure CM fraction with minimal NM contamination. In contrast, the NM fraction expressed *Col1α1* at high levels that were indistinguishable between groups (Fig. 5A), although a small increase was noted after AngIV treatment when relative cell contribution was taken into consideration (supplementary material Fig. S3A). However, the lack of significant increase in collagen indicates that this aspect does not present until later in the progression towards pathological remodelling. Similar to the *Col1α1* profile, the CM population did not express *Tgfβ* (Fig. 5B). However, the NM fraction showed a progressive increase in *Tgfβ* in both the Tg and AngIV groups (Fig. 5B, supplementary material Fig. S3A), suggesting that increased *Tgfβ* production precedes collagen, consistent with causative but delayed action in this respect. TGFβ has been previously reported to promote synthesis of vascular endothelial growth factor α (VEGFα) (Nakagawa et al., 2004). VEGFs are essential regulators of vasculogenesis, angiogenesis and vessel maintenance during embryonic development and adulthood (Zentilin et al., 2010) that are known to be upregulated during pathological remodelling to compensate for increased oxygen demand. We found that CM and NM fractions expressed *Vegfα* at similar levels in WT hearts (Fig. 5C, supplementary material Fig. S3A). However, although a slight tendency towards increasing *Vegfα* expression was detected in the CM population, the NM population exhibited a reciprocal and more substantial downward trend leading to a substantial differential between CM- and NM-derived expression after AngIV treatment.

### Pro-inflammatory genes are dynamically regulated in NM cells of HART mice

Inflammation has been closely associated with heart disease (Shinde and Frangogiannis, 2014; Turner, 2014), and CD68 staining used in this study demonstrated significant macrophage infiltration in Tg myocardium following AngIV stimulation, but not before. We therefore analysed the inflammatory gene signature of the NM population in WT mice, and in Tg mice before and after AngIV treatment.

Tenascin-C (TNC) is associated with cellular injury and inflammation, and has many cellular functions, with roles in embryogenesis, wound healing and cancer progression (Imanaka-Yoshida et al., 2004). Consistent with downregulation of *Vegfα* from Tg NM cells, *Tnc* expression was also decreased (Fig. 5D, supplementary material Fig. S3B). However, in contrast to *Vegfα*, *Tnc* returned to WT levels following AngIV treatment. The pro-inflammatory cytokines *IL-6* and *IL1-β* were similarly decreased in the Tg group, with only *IL-6* returning to normal levels upon AngIV stimulation (Fig. 5D, supplementary material Fig. S3B). The chemokine *Cxcl2* showed a similar profile, with a small initial decrease in the Tg group that was reversed following AngIV stimulation (Fig. 5D, supplementary material Fig. S3B).

Of particular note, all of the inflammation-associated genes analysed were initially repressed in Tg animals that have cardiac dysfunction without evidence of structural myocardial remodelling. Even under conditions that lead to early stages of fibrosis and macrophage infiltration, a substantial inflammatory response was not initiated. The evidence therefore suggests that inflammatory responses frequently associated with the remodelling process are late-stage secondary responses. More importantly, our data suggest that there is an initial inhibition of pro-inflammatory signalling from the NM population in the presence of a functionally compromised CM population, possibly in response to upregulation of TGFβ (van Nieuwenhoven and Turner, 2013). This compensatory response is reversed when the CM population is stressed to a critical level.

### Differential expression of genes associated with CM dysfunction

To get to the heart of initiating events driving CM dysfunction in HART mice, rather than the frequently reported secondary responses from other cell types in the myocardium, we investigated CM-expressed genes that are commonly associated with CM dysfunction. This includes activation of immediate early genes (Lee et al., 1988), re-expression of foetal genes (Izumo et al., 1988) and changes in expression of contractile and calcium handling proteins (Braunwald and Bristow, 2000; Miyata et al., 2000).

Upregulation of atrial natriuretic factor (ANF) is commonly used as a marker for hypertrophy. ANF exhibits diuretic, natriuretic and vasorelaxant effects and is responsible for maintaining blood pressure and natriuresis (Lucas et al., 2010). ANF also reduces fibrosis by inhibition of collagen synthesis from cardiac fibroblasts (Redondo et al., 1998). Not unexpectedly in view of the observed lack of CM hypertrophy, and consistent with the pattern seen for pro-inflammatory genes in the NM fraction, *Anf* expression was found to be significantly reduced in CM cells of the Tg group (Fig. 5E). Following AngIV treatment, *Anf* levels remained low (supplementary material Fig. S3C). Thus, ANF does not play a role in induction of dysfunction in the HART model.

Recently, it has been suggested that, following activation of AT1R, collagen can directly modulate the calcium dynamics and electrical activities of atrial CM cells by increasing expression of the sarcoplasmic reticular ATPase *Serca2α* and Thr17-phosphorylated phospholamban, with no alteration in expression of *Ryr2* (Lu et al., 2011). In contrast, *Serca2α* was shown to be downregulated in cardiac hypertrophy, resulting in impaired diastolic and systolic function (Houser and Margulies, 2003). Consistent with this, Iijima et al. (1998) demonstrated that *Serca2a* is downregulated after myocardial infarction, correlating with increased AT1R expression. Zentilin et al. (2010) also found *Serca2α*, *Ryr2* and *Pgc1α* to be significantly decreased following myocardial infarction, accompanied by hypertrophy and dysfunction. In our HART model, we found that *Serca2α* levels dropped only slightly, whereas *Ryr2* showed a more substantial tendency to decrease (Fig. 5E, supplementary material Fig. S3C). Taken together with the tendency towards reducing levels of *Vegfα* driven mainly by the NM population, dysregulation of *Ryr2* might play a part in initiation of CM dysfunction in our model.

Of greater interest, however, is PGC1α, a key regulator of mitochondrial bioenergetics in the heart. Cardiac-specific repression of PGC1α results in downregulation of mitochondrial enzymes, morphological impairment and loss of mitochondria (Czubryt et al., 2003). Furthermore, in chronic heart failure, PGC1α is downregulated and correlates strongly with muscle oxidative capacity (Garnier et al., 2003). We therefore examined whether the contractile dysfunction observed in the Tg HART animals might be directly attributable to modulation of PGC1α. Notably, expression was significantly decreased in the Tg group, and further decreased following AngIV treatment (Fig. 5E, supplementary material Fig. S3C), suggesting that compromised CM mitochondrial function might play a part in the initiation of CM dysfunction. This warrants further detailed investigation.

## DISCUSSION

**Fig. 6.**
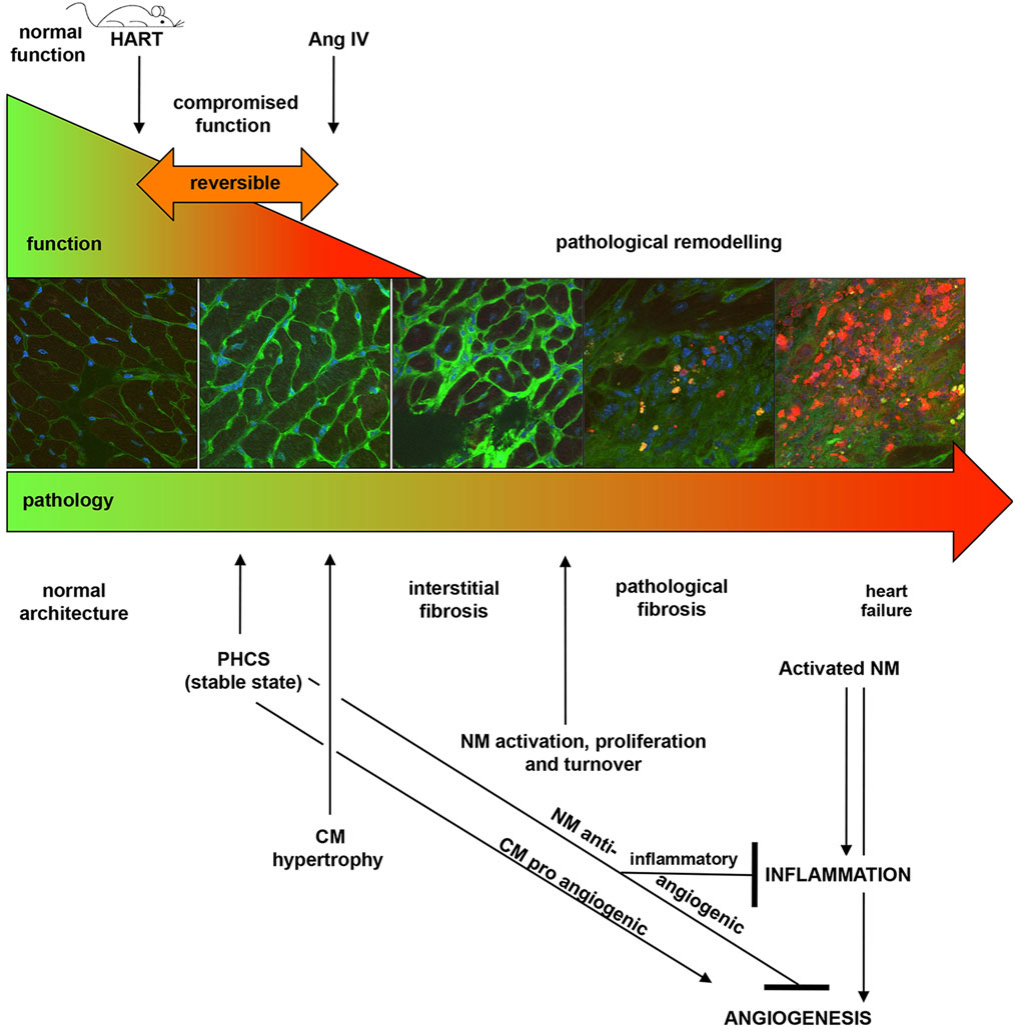
**Summary schematic of stepwise progression of heart disease in response to CM-specific AT1R activity.** From left to right: normal pathology and function are indicated in green; compromised function and pathological remodelling are indicated in red. Basal HART expression causes CM dysfunction with no impact on pathology; this dysfunction is associated with compromised mitochondria and an attempt to increase angiogenic signalling from the distressed CM cells. This correlates with dampened expression of pro-inflammatory and angiogenic genes from the NM population, leading to a reversible ‘pre-hypertrophy compensated state’ (PHCS). Treatment with AngIV, however, rapidly induces CM hypertrophy and sporadic cell death leading to state reversal, activation of cardiac fibroblasts, macrophage infiltration and a further decrease in angiogenic response. This rapidly leads to uncontrolled cell turnover in the NM population, driving interstitial fibrosis and ultimately heart failure. PHCS represents an ideal state for therapeutic intervention.

The RAS has been the subject of intense investigation for decades. However, the full complexity and cell-type-specificity of its involvement in initiating stages of heart disease remains enigmatic. Although numerous studies have exploited alterations in components of the RAS, none has yet clearly depicted initiating events. Here, we have delineated stepwise progression of the earliest stages using conditional transgenic HART mice, in which expression of a human AT1R is activated exclusively in fully differentiated adult CM cells, with no changes in blood pressure. Our data show that cardiac hypertrophy does not develop initially; rather, cardiac dysfunction is a prominent initiating event. It is noteworthy that even in a state of dysfunction the different cellular compartments of the heart apparently interact to prevent hypertrophic, inflammatory or angiogenic responses. Thus, we have referred to this condition as a pre-hypertrophic compensated state (PHCS) that is remarkably stable and reversible (Fig. 6).

Upon further stimulation of the receptor, without blood pressure change, dysfunction is amplified, CM cells become hypertrophic and sporadic fibrosis ensues. Once this process commences it becomes very difficult to separate cause from consequence. The results are consistent with a previously reported model of AT1R overexpression that displayed CM hypertrophy with enhanced collagen deposition, myolysis and cardiac remodelling (Paradis et al., 2000). At 6 months of age, these mice were reported to exhibit cardiac hypertrophy and ventricular contractile dysfunction (Rivard et al., 2008). However, in young adults there was no evidence of hypertrophy, but the CM cells exhibited both decreased fractional shortening and contractility underlying the dysfunction (Rivard et al., 2011). A further study was reported in which AT1Rs were overexpressed in CM cells on an *AngII*-null background (Yasuda et al., 2012). Constitutive AT1R activity was found to contribute to progressive cardiac dysfunction and remodelling without AngII stimulation. This is consistent with reports that the AT1R can exhibit spontaneous activity, through utilisation of the receptor’s potential energy (Karnik and Unal, 2012; Parnot et al., 2002). Taken together, the data show that increased AT1R activity in CM cells will have serious detrimental consequences if left unchecked.

Whole heart enlargement is primarily caused by hypertrophy of pre-existing CM cells. In progressive heart disease, as well as during natural aging, the rate of CM death increases and, as a compensatory response, CM hypertrophy is initiated (Nadal-Ginard et al., 2003). Two distinct mechanisms lead to cell death: apoptosis and/or oncosis. During apoptosis the cell shrinks and is removed by neighbouring cells with limited influence on tissue morphology and without collagen accumulation. In contrast, oncosis is accompanied by an inflammatory response, vessel proliferation, macrophage infiltration and fibroblast activation leading to collagen deposition and scar formation (Nadal-Ginard et al., 2003). Even a moderate rate of CM death can gradually lead to a reduction in myocardial mass and result in chronic heart failure. In our model, cell death was observed in the heart under conditions where the transgenic receptor was unstimulated. Notably, death was limited to the NM population and was actually associated with downregulation of inflammation-associated genes, suggesting that regulated apoptosis is responsible for natural turnover of NM cells. IL-6 has been correlated with increased risk of LVH, dilatation, dysfunction and fibrosis (Birner et al., 2007; Yan et al., 2010), so reduced expression in the dysfunctional heart strongly supports an active attempt to limit pathological remodelling. TNC showed a similar reduction in expression. This pro-angiogenic molecule (Ballard et al., 2006) is upregulated in many pathological conditions, including myocardial infarction, myocarditis, hibernating myocardium and dilated NC cardiomyopathy (Imanaka-Yoshida et al., 2001; Sato et al., 2002; Tamura et al., 1996). It has also been reported to modulate proliferation and migration (Stepp and Zhu, 1997; Yoshimura et al., 1999), playing an important role in wound healing (Tamaoki et al., 2005).

Following stimulation with AngIV, the rate of cell death in the NM population increased, CM death was also observed, and expression of inflammatory markers began to increase. Although none of the genes tested were expressed above WT levels, it is possible that this renewed expression was highly focussed within areas of remodelling. The changes coincided with infiltration by macrophages, suggesting that stimulation of an inflammatory response might be in response to CM oncosis rather than apoptosis. However, it remains undetermined whether the macrophage infiltrates occurred in response to dying CM cells, or to the increased rate of NM cell turnover. Indeed, macrophages were generally only observed in areas of significant fibroblast accumulation, and not around isolated CMs in healthy areas of the myocardium, suggesting that progressive infiltration and accumulation might be exacerbated by events occurring at sites populated by NM cells. In any event, it was notable that little difference was detected in *Col1α1* at any stage, suggesting that the changes observed reflect early events that precede progressive cardiac remodelling. This is entirely consistent with the observed ability for the heart to recover function upon suppression of HART expression, demonstrating a ‘window of opportunity’ for successful treatment of cardiac dysfunction by suppression of local RAS activity, prior to induction of pathological remodelling (Fig. 6).

In contrast to all other genes tested, *Tgfβ* expression was upregulated in NM cells at the earliest stages of remodelling. This complex signalling molecule has been reported to possess anti-inflammatory and anti-proliferative activity (Redondo et al., 2012), which is consistent with our observations in the unstimulated Tg hearts. However, inhibition of *Tgfβ* signalling in the pressure overloaded heart has also been found to attenuate myofibroblast proliferation and collagen deposition (Lucas et al., 2010). Our observation of an increase in proportion of NM cells relative to CM cells following AngIV stimulation supports a pro-proliferative role in this context.

An increase in CM-specific *Vegfα* expression was detected in transgenic animals both with and without AngIV treatment. However, there was a responsive decrease in the NM cells consistent with a secondary response to rebalance the level of pro-angiogenic gene expression within the myocardium. VEGFs have been previously shown to induce a gene expression programme consistent with compensatory cardiac hypertrophy, by elevation of *Anf*, *Bnp*, *αMHC*, *Serca-2α*, *Ryr2* and *Pgc1α*, and by inhibition of *βMHC* and skeletal alpha-actin (*sk α-act*) (Zentilin et al., 2010). In HART mice, expression of *Anf*, *Ryr2* and *Pgc1α* were observed to decline in the CM population. This is not surprising given the significant reduction in *Vegfα* expression from the HART NM population. We suggest that the HART model represents a very early stage of pre-hypertrophy in which VEGF signalling has not stabilized, the decrease in the NM population representing an initial mechanism to prevent fibrosis by inhibition of extracellular matrix. It has been previously suggested that VEGF is an essential mediator of AngII-induced vascular inflammation and remodelling, but is not involved in AngII-induced cardiac hypertrophy (Zhao et al., 2004). Our observation of a tendency towards increased macrophage infiltration and sporadic fibrosis in HART mice following AngIV treatment, at a time when *Vegfα* expression is further suppressed from the NM population, suggests that VEGFα is not a principal mediator of this response.

Upon activation of the intracellular CM RAS by AngIV, dysfunction and dilatation were intensified, leading to hypertrophy, increased cell death, macrophage infiltration and sporadic fibrosis. The results are consistent with an early transition out of a compensated pre-hypertrophic state towards fixed pathological remodelling. We suggest that alterations in mitochondrial energetics might represent a suitable therapeutic target that warrants detailed investigation.

## MATERIALS AND METHODS

### HART mouse model

Animal work was carried out with ethical approval (University of Leeds, UK) under UK Home Office licence. The study utilised male HART mice that in adulthood express a constitutively active mutant form of human AT1R in differentiated cardiomyocytes, through action of a cell-type-specific tetracycline transactivator (Ainscough et al., 2009). The experiments thus utilised double-transgenic (Tg) animals that harboured both *hAT1R*-*lacZ* responder and *αMHC-tTA* activator transgenes (Tg group), as well as sex- and aged-matched wild-type littermate controls (WT group). WT mice harboured either transgene independently, or neither transgene. The mice were bred on Dox (125 μg/ml) and weaned onto normal drinking water at 1 month. Previous results showed that HART is maximally activated within 2 weeks of Dox withdrawal, and high-level expression continues into adulthood. All mice were analysed at 5 months, unless otherwise stated. Some transgenic animals (Tg) were infused with AngIV continuously at 5 µg/kg body weight/min for 4 weeks via a subcutaneously implanted miniosmotic pump. WT mice were used as controls against Tg mice, whereas Tg and Tg mice treated with saline for 4 weeks were used as controls against Tg mice treated with AngIV for 4 weeks. No significant differences were observed between Tg and Tg-saline mice.

### Ventricular function

Millar catheter analysis was performed as previously described (Ainscough et al., 2009). Briefly, mice were anaesthetised with isoflurane and the right carotid artery exposed. A 1.4 F miniature pressure-volume catheter (SPR-839, Millar Instruments, USA) was inserted through the right carotid artery into the ascending aorta and then into the left ventricle via the aortic valves. This was connected to an MPVS-300 (Millar Instruments) and data were recorded using AD instruments Chart software (Chart 5 Pro). PV loop analysis was performed using PVAN 3.6.

### Cardiac weight index (CWI)

Body weight was recorded, then the heart removed and cleaned with ice-cold PBS. Atria were removed and assayed for *lacZ* activity to confirm genotype (Ainscough et al., 2009). The ventricles were cleared of blood, blotted, weighed and snap-frozen in liquid nitrogen. Tibia were also removed, cleaned and measured. Ventricular weight to tibia length ratio (mg/mm) was recorded as CWI. Ventricle to body weight ratio (mg/g) was also determined.

### Cardiac-cell isolation

CM and NM cells were separated into fractions using a modified Langendorff apparatus. All buffers were pre-warmed to 37°C unless otherwise stated. Males were euthanized, hearts excised, cannulated via the aorta onto a Langendorff apparatus, and perfused with dissociation buffer (130 mM NaCl, 5 mM KCl, 25 mM Hepes, 0.5 mM MgCl_2_, 0.33 mM monobasic NaH_2_PO_4_, 22 mM D-glucose, 3 mM Na pyruvate) containing 100 mM EGTA until cleared of residual blood. The heart was then perfused with 25 ml dissociation buffer containing 144 U/ml collagenase (Worthington type-2) and 50 µM CaCl_2_. The ventricles were placed in 12.5 ml dissociation buffer supplemented with 144 U/ml collagenase, 100 µM CaCl_2_, 1% BSA at room temperature, cut into small pieces and gently agitated by repeat pipetting. The cell suspension was filtered through nylon gauze to remove remaining tissue clumps, then processed for isolation of cardiomyocyte (CM) and non-myocyte (NM) fractions. A loose CM pellet was first settled by gravity, then washed in 10-ml ice-cold digestion buffer containing 250 µM CaCl_2_ and 1% BSA.

### RNA extraction, cDNA synthesis and qPCR

For RNA isolation, CM cells were further purified away from remaining NM cells by gentle resuspension and centrifugation in ice-cold PBS (3×1 min) at 500 rpm at 4°C, then resuspended in 1 ml TRI-reagent (Ambion, UK). The remaining NM-cell-containing supernatant from the initial CM settling was further cleared of CM cells by sequential centrifugation (4×1 min) at 500 rpm at 4°C until no CM pellet was visible. The remaining supernatant was centrifuged at 1500 rpm for 10 min to pellet the NM population, which was resuspended in TRI-reagent for RNA isolation following the manufacturer's instructions. For whole heart RNA, ten 30-µm cryo-sections were cut using a Leica cryostat, homogenised in 1 ml TRI-reagent, then processed as before. All RNA samples were treated with TURBO DNase (Ambion, UK) and reverse transcribed into cDNA with Superscript III (Invitrogen, UK) using random hexamers (Sigma). For qPCR analysis, cDNAs were added to reactions containing Taqman PCR master mix, gene-specific primer-probe sets for *Col1α1*, *Tgfβ*, *Vegfα*, *Tnc*, *IL-6*, *IL-1β*, *Cxcl-2*, *Anf*, *Serca2α*, *Ryr2*, *Pgc1α* and *Gapdh* (Applied Biosystems), and amplified using an ABI-7500-PCR system. All samples were normalised to *Gapdh.* Relative expression was calculated as 2^−ΔCT^×100 and presented as % *Gapdh*.

### Histology for cell counts and macrophage infiltration

10-µm heart cryo-sections were mounted on poly-L-lysine-coated slides and fixed with 4% paraformaldehyde for 20 min, washed with PBS, incubated with rhodamine-linked wheat germ agglutinin (WGA; 1:1000; Vector Laboratories, UK) for 2 h, washed again with PBS and mounted in VectorShield with DAPI (Vector Laboratories, UK). To assess macrophage infiltration, fixed heart cryo-sections were permeabilized with 0.5% Triton X-100 for 20 min, then blocked with 10% horse serum in PBS for 1 h prior to incubation in 10% horse serum/PBS containing 1/100 rat anti-CD68 antibody (Abcam, UK) and 1/1000 WGA for 2 h. The sections were rinsed twice with PBS, incubated with 1/1000 anti-rat IgG-FITC secondary antibody (Abcam, UK) in 10% horse serum/PBS for 1 h, washed three further times with PBS and mounted in VectorShield with DAPI. High-resolution thin-section (0.5 µm) images were taken by confocal microscopy using a Zeiss LSM Axiovert 200M microscope fitted with a 63× water objective, Zeiss LSM510 software and Adobe Photoshop CS. Images in Fig. 1 were taken using a Zeiss AxioImager epifluorescence microscope with AxioVision digital image processing software. CM cross-sectional area counts were averaged from >four animals per group, five fields of view per animal (40× objective) from healthy areas of the left ventricle.

### Cell death

Click-iT TUNEL assay was performed to assess DNA fragmentation in left-ventricular tissue according to the manufacturer's instructions. Briefly, heart cryo-sections were fixed in 4% paraformaldehyde and permeabilized with 0.25% Triton X-100. 100 µl of TdT reaction buffer was added to the sections for 10 min, followed by the TdT reaction cocktail for 1 h at 37°C in a humidified chamber. The sections were washed with 3% BSA and incubated in Click-iT reaction cocktail for 30 min. FITC-conjugated WGA was added for 2 h, followed by Hoechst 33342 (1/5000) for 15 min. The sections were mounted in VectorShield and analysed by confocal microscopy as before. % TUNEL-positive CM and NM cells were determined from a total of ten images (63× magnification) per animal.

Detection of cells undergoing apoptosis was performed using Alexa-Fluor-488-conjugated anti-cleaved caspase-3 (Cell Signaling Technology). Heart cryo-sections were fixed in 4% paraformaldehyde for 20 min, then permeabilized with 0.5% Triton X-100 for 20 min. The sections were blocked with 5% normal goat's serum for 1 h prior to overnight incubation with anti-cleaved caspase-3 (1:50) in a humidified chamber at 4°C. The heart sections were rinsed with PBS, and rhodamine-conjugated WGA (1:2000) applied for 2 h. The sections were then rinsed with PBS (3×5 min), and mounted in VectorShield with DAPI. The stainings were repeated twice, on a total of nine individual sections per genotype.

For positive detection of cleaved caspase-3, mouse NIH3T3 fibroblasts were treated with 1 µm staurosporine (Sigma) for 5 h at 37°C and the staining was performed as described above. Fluorescent images were taken with a Zeiss Axiovert microscope fitted with a 63× oil-immersion objective and an AxioCam camera, using Openlab software with pre-determined standardized settings (Improvision). Images were subsequently manipulated using Adobe Photoshop to facilitate multicolour image overlay.

### Statistical analysis

Data are presented as mean±s.e.m. Unless otherwise stated, data samples were analysed by unpaired *t*-test using Graph Pad Prism v5.01 (GraphPad Software Inc., USA). A value of *P*≤0.05 was considered significant. (*P≤0.05, **P≤0.01 or ***P≤0.001).

## Supplementary Material

Supplementary Material
