# Supplementary material for: A state of reversible compensated ventricular dysfunction precedes pathological remodelling in response to cardiomyocyte-specific activity of angiotensin II type-1 receptor in mice
Source: Dis Model Mech. 2015 Aug 1;8(8):783–94. doi: 10.1242/dmm.019174 (PMC4527284; doi:10.1242/dmm.019174)
Supplement: Supplementary Material [file supp_8_8_783__index.html]

Supplementary Material 

# A state of reversible compensated ventricular dysfunction precedes pathological remodelling in response to cardiomyocyte specific Angiotensin II type-1 receptor activity

## DMM019174 Supplementary Material

- Supplementary Material
