# Supplementary material for: A state of reversible compensated ventricular dysfunction precedes pathological remodelling in response to cardiomyocyte-specific activity of angiotensin II type-1 receptor in mice
Source: Dis Model Mech. 2015 Aug 1;8(8):783–94. doi: 10.1242/dmm.019174 (PMC4527284; doi:10.1242/dmm.019174)
Supplement: Supplementary Material [file supp_019174_DMM019174supp.pdf]

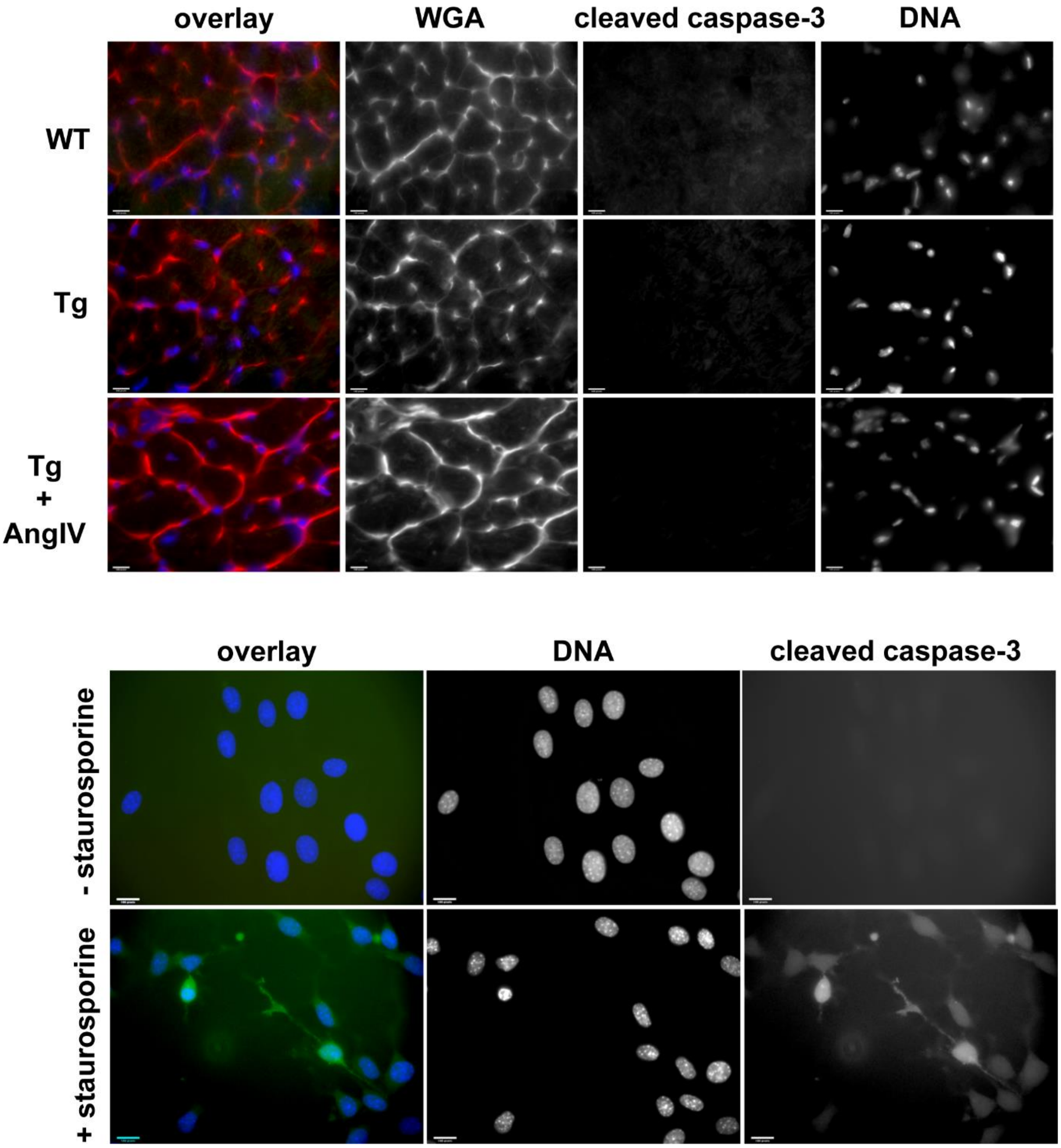

Supplementary Figure 1 Cleaved caspase-3 analysis. Representative images (upper panels) of sections of left ventricle taken from WT and Tg mice, and Tg mice following stimulation with AngIV for 4-weeks, and (lower panels) cultured NIH3T3 cells exposed to staurosporine at 0 μm (negative control) and 1 μm (positive control) for 5-hours. All samples are stained with AlexaFluor conjugated anti-cleaved Caspase-3 (green) to detect apoptotic cells, and Dapi (blue) to detect cell nuclei. Heart sections are also stained with WGA (red) to show cell membranes. Scale bar-10μm.

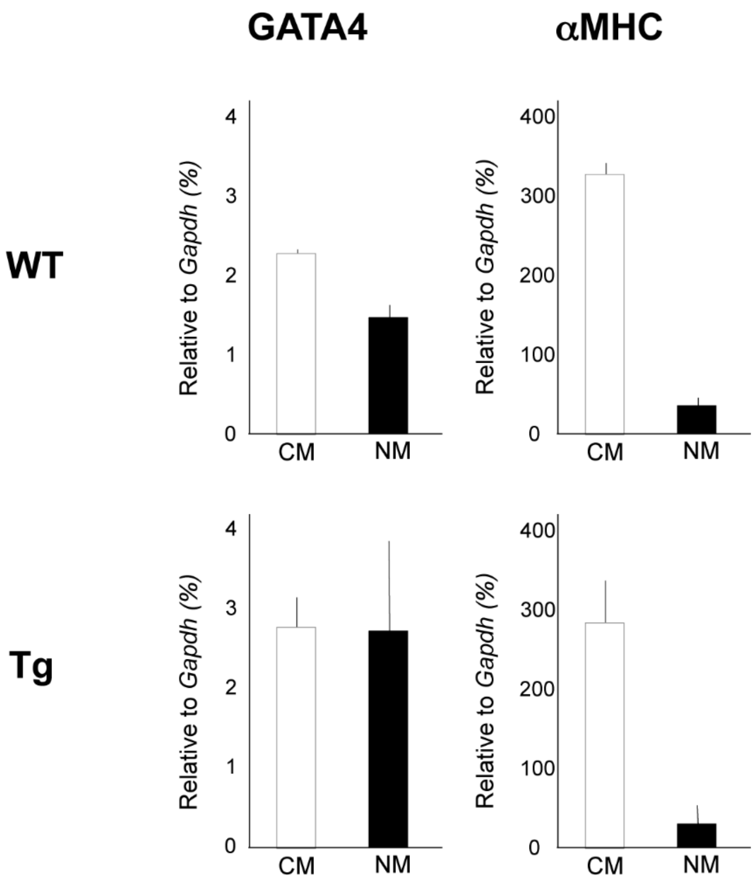

Supplementary Figure 2 Cardiac specific gene expression analysis, relative to *Gapdh,* of CM and NM cellular fractions of WT and Tg mouse hearts. GATA4 is expressed at similar levels in each fraction, while αMHC expression predominates in the CM fraction.

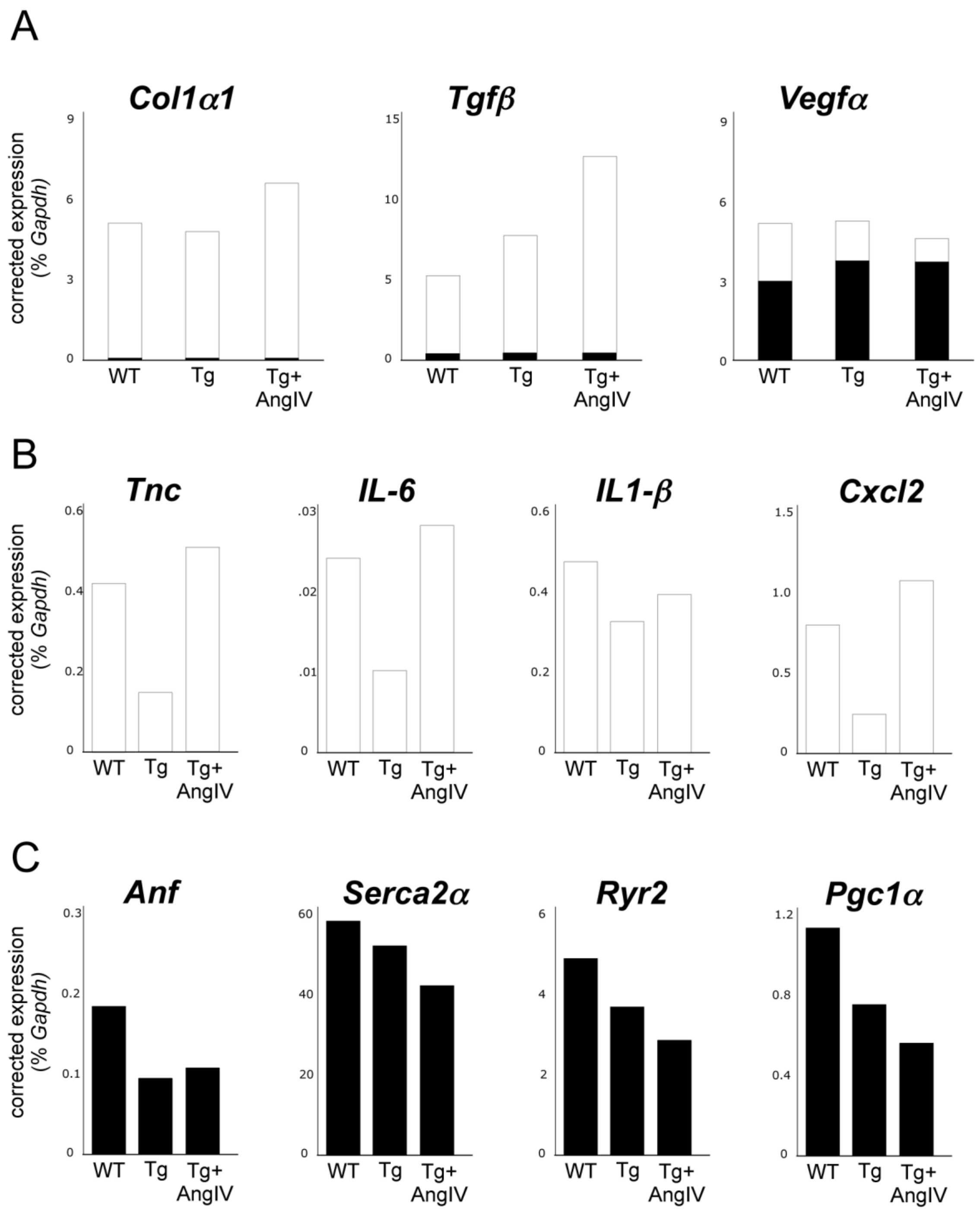

Supplementary Figure 3 Gene expression analysis presented in Fig 5 adjusted to take account of the relative proportion of CM and NM cells in hearts of different genetic and treatment status, as determined using the parameters considered in Fig 3. (A) Proportional expression of genes in CM (black) and NM (white) illustrate dynamic change in total expression in the heart, and cell type from which expression is derived. (B) 'Corrected' NM specific gene expression. (C) 'Corrected' CM specific gene expression.
